# Supplementary material for: Families with BAP1-Tumor Predisposition Syndrome in The Netherlands: Path to Identification and a Proposal for Genetic Screening Guidelines
Source: Cancers (Basel). 2019 Aug 4;11(8):1114. doi: 10.3390/cancers11081114 (PMC6721807; doi:10.3390/cancers11081114)
Supplement: Supplementary file 1 [file cancers-11-01114-s001.pdf]

Supplementary Materials

Families with BAP1-Tumor Predisposition Syndrome in the Netherlands: Path to Identification and a Proposal for Genetic Screening Guidelines

Cindy Chau, Remco van Doorn, Natasha M. van Poppelen, Nienke van der Stoep, Arjen R. Mensenkamp, Rolf H. Sijmons, Barbara W. van Paassen, Ans M.W. van den Ouweland, Nicole C. Naus, Annemieke H. van der Hout, Thomas P. Potjer, Fonnet E. Bleeker, Marijke R. Wevers, Liselotte P. van Hest, Marjolijn C.J. Jongmans, Marina Marinkovic, Jaco C. Bleeker, Martine J. Jager, Gregorius P.M. Luyten and Maartje Nielsen

Table S1. Malignancies found in proband, tested non-proband carriers and untested members from families with germline variants in *BAP1*.

Family Identifier	Tumor Proband (Age)	Number of Nonproband Variant Carriers	Tumor in Nonproband Variant Carriers (Age)	Number of Untested Relatives	Tumor in Untested Relatives
NL-1	UM (67)	9	- UM (65), GI cancer (?)<br>- UM (61), NMSC (59)<br>- NMSC (65), Breast cancer (55), Endometrial cancer (66)<br>- GI cancer (?)	12	UM, Breast cancer
NL-2	BIN x2 (39, 39)<br>NMSC (39)	7	- CM (41)<br>- MMe pl (72), GI cancer (55), Breast cancer (59)<br>- MMe pl (63)<br>- MMe pl (64), fibrosarcoma (64)<br>- NMSC (49), Warthin tumor (?)<br>- NMSC (48)<br>- Breast cancer x2 (52, 55)	61	UM, CM x3, MMe pl x3, MMe pt x2, NMSC x3, Skin cancer x10, GI cancer, Breast cancer, Multiple myeloma
NL-3	BIN x5 (29, 29, 29, 29, 29)	0	-	11	CM, NMSC x4, Ewing sarcoma, Hodgkin lymphoma
NL-4	MMe pt (39)	1	Lung cancer	18	CM, MMe pl, MMe pt, NMSC x2, Ocular tumor, Breast cancer, Unknown tumor
NL-5	UM (66)<br>NMSC (66)<br>Meningioma (44)<br>Vestibular schwannoma (51)	0	-	36	UM, CM, RCC, Meningioma, Skin tumor, Lung cancer x4, GI cancer, Mouth cancer
NL-6	UM (30)	8	- CM (29)<br>- MMe pt (?)<br>- NMSC x9 (56, 60, 62, 62, 63, 63, 64, 65, 66), Prostate cancer (65), Urothelial cell cancer (66)	19	CM, RCC, Ocular tumor, Skin tumors, Breast cancer, Leukemia

NL-7	BIN x2 (22, 22)	0	-	N/A [a]	N/A
NL-8	CM (49)<br>Conjunctival melanoma (44)<br>Lung cancer (54)	0	-	39	CM, RCC, NMSC, Lung cancer x2
NL-9	UM (57) - iris<br>MMe pl (61)<br>RCC (61)<br>NMSC (57)<br>B-cell lymfoma (58)	0	-	20	RCC, Prostate cancer, Pancreatic cancer, Unknown malignancy
NL-10	BIN x2 (20, 26)<br>NMSC (27)	1	- NMSC x3 (57, 59)	19	Urothelial cell cancer, Tongue cancer, Unknown metastatic cancer in liver
NL-11	BIN (55)<br>Breast cancer (48)	4	- NMSC x5 (48, ?)<br>- CM (51), NMSC x8 (45, 47, 54, 54, 57, 57, 57)	30	UM, Skin cancer, Bone cancer, Unknown malignancy x5
NL-12	UM (53)<br>CM (56)<br>NMSC (38, 52)	3	- MMe pl (54), RCC (58)	20	CM, Lung cancer x2, GI cancer, Breast cancer
NL-13	BIN x4 (15, 15, 18, 18)	0	-	N/A[a]	N/A
NL-14	CM x2 (23, 27)<br>NMSC x2 (50, 53)	5	- CM (49), NMSC x36 (?), Schwannoma (52)<br>- CM x2 (70, 83), NMSC x6 (50, 75, 76, 79, 79, 80)<br>- NMSC (50)<br>- NMSC (40)	26	CM x3, Metastatic (liver) melanoma of unknown origin, NMSC x3, Lung cancer, Non-hodgkin lymphoma
NL-15	BIN x2 (21, 25)	4	-	12	UM, Unknown malignancy x2
NL-16	CM (65)	0	-	25	CM x3, RCC, Lung cancer x2, Breast cancer, Liver cancer, Brain tumor, Abdominal malignancy
NL-17	BIN x2 (14, 14)	1	- CM (44)	2	RCC, Meningioma, Prostate cancer
NL-18	UM (72)<br>Hepatocellular carcinoma (68)	5	- MMe pl (61), NMSC x3 (67, 67, 67), Hepatocellular carcinoma (53)<br>- Hepatocellular carcinoma (51)<br>- Breast cancer (51)<br>- NMSC x2 (58)	31	MMe pl, MMe pt, Ocular tumor x2, GI tumor, Liver tumor, Brain tumor, Thyroid tumor
NL-19	CM (44)	1	-	29	UM, NMSC, Lung cancer, Prostate cancer x2
NL-20	CM (45)	0	-	24	CM x3, Lung cancer x2, GI cancer, Liver cancer, Pleuritis carcinomatosa -primairy likely lung or thyroid
NL-21	UM (44)<br>CM (55)	2	-	89	CM, RCC, Lung cancer x4, Breast cancer, Bone cancer x2, Throat cancer, Abdominal tumor x2, Unknown malignancy
NL-22	CM (47)<br>NMSC x7 (54, ?)<br>Prostate cancer (55)	1	- CM (23)	10	CM, NMSC >2, Lung cancer

UM: uveal melanoma, CM: cutaneous melanoma, MMe: malignant mesothelioma, pl: pleural, pt: peritoneal, RCC: renal cell cancer, BIN: *BAP1*-inactive nevus, NMSC: non-melanoma skin cancer (mostly basal cell carcinomas), GI: gastro-intestinal.

Supplementary Data 1.

RNA Analysis of *BAP1* Variant c.122+5G>C (NL-17)

Method

RNA was isolated from cultured lymphocytes in the presence of nonsense mediated decay-inhibitor cycloheximide essentially as described by Vreeswijk et al. [35]. Complementary DNA (cDNA) was sequenced by PCR using a forward primer in exon 1 (GAATAAGGGCTGGCTGGAG) and a reverse primer in exon 4 (GGACGTATCATCCACCAAGG) followed by Sanger sequencing.

Sequencing chromatogram (forward strand) of the cDNA shows the transition of exon 2 to exon 3 and to exon 4 of the mutated allele, which demonstrates skipping of exon 3 at the mutated allele.

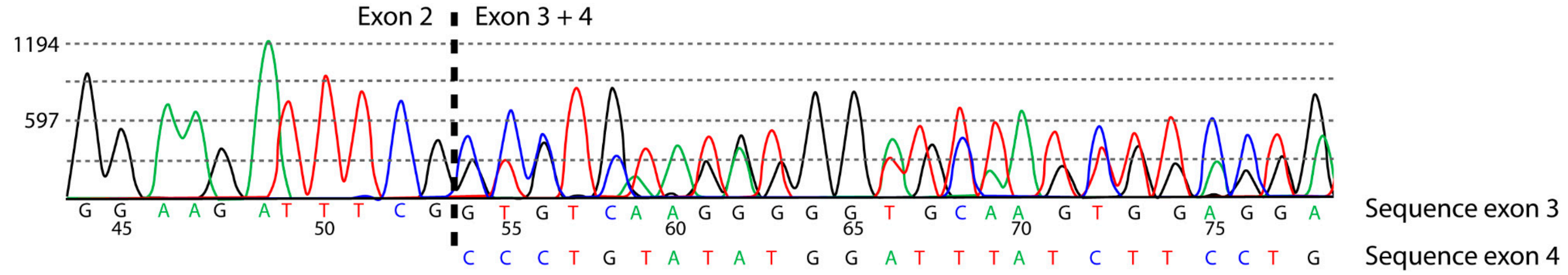

The variant leads to a skip of exon 3 from the transcript. The nomenclature of the found change in the RNA is r.68_122del (p.(Gly23fs)). Although the data is suggestive for a complete deletion, it cannot be excluded that some full-length transcript is still transcribed from the mutant allele; the variant is therefore classified as likely pathogenic.

© 2019 by the authors. Licensee MDPI, Basel, Switzerland. This article is an open access article distributed under the terms and conditions of the Creative Commons Attribution (CC BY) license (http://creativecommons.org/licenses/by/4.0/).
